# It’s A Pain in The Neck: Case Report of Bedside Diagnosis of Unilateral Neck Swelling

**DOI:** 10.5811/cpcem.53021

**Published:** 2026-04-29

**Authors:** Maxx F. Hotton, Kevin R. Roth, Kristine L. Schultz

**Affiliations:** * Lehigh Valley Health Network, Department of Emergency and Hospital Medicine, Allentown, Pennsylvania; † Lehigh Valley Health Network / USF Morsani College of Medicine, Department of Emergency and Hospital Medicine, Division of Point of Care Ultrasound, Allentown, Pennsylvania

**Keywords:** Lemierre syndrome, internal jugular thrombus, point-of-care ultrasound, case report

## Abstract

**Introduction:**

Lemierre syndrome is a rare but potentially severe thrombophlebitis of the internal jugular vein. It most often presents after oropharyngeal infection, likely stemming from anaerobic bacteria, commonly *Fusobacterium necrophorum*. The potential severity of this condition underscores the importance of early and accurate diagnosis. The gold standard diagnosis relies on computed tomography and blood cultures; however, point-of-care ultrasound offers a rapid and cost-effective tool.

**Case Report:**

A 58-year-old woman with chronic obstructive pulmonary disease, migraines, and recent dental extractions presented with two days of worsening right-sided neck pain and swelling. She denied fever, chills, or recent upper respiratory symptoms. Examination revealed a tender anterior neck mass without airway compromise. Point-of-care ultrasound demonstrated a 1.22 x 1.80 centimeters hyperechoic intraluminal mass within the right internal jugular vein with surrounding cobblestone edema; the external jugular vein and carotid artery were normal. Computed tomography imaging confirmed the diagnosis. Laboratory studies were unremarkable. Blood cultures were obtained, and empiric intravenous beta lactamase-resistant antibiotics was initiated. Anticoagulation was considered but not started.

**Conclusion:**

A delayed diagnosis of Lemierre syndrome is common, as its early presentations are commonly nonspecific. The classic triad is a recent oropharyngeal infection, internal jugular vein thrombosis, and septic emboli. The importance of early diagnosis and treatment cannot be overstated, as it improves outcomes while reducing costs and radiation exposure for the patient. Point-of-care ultrasound is a valuable first-line imaging modality for Lemierre syndrome. Its use in patients with symptoms such as unexplained neck swelling and tenderness can facilitate timely diagnosis and treatment, thereby averting serious adverse outcomes.

## INTRODUCTION

Lemierre syndrome is a thromboembolic and septic complication most commonly caused by *Fusobacterium necrophorum* following oropharyngeal infections.^1^,^2^ The classic triad includes recent oropharyngeal infection, internal jugular vein thrombosis, and metastatic septic emboli.^1^–^3^ While traditionally diagnosed through computed tomography (CT) imaging and blood cultures,^3^,^4^ the importance of point-of-care ultrasound (POCUS) has increased in emergency medicine due to its rapid, cost-effective bedside utility.^5^–^7^ Delayed diagnosis of Lemierre syndrome is associated with poor outcomes such as septic shock, intensive care unit admission and, in one study, a 30-day mortality rate of 2%.^8^ Additionally, analysis of 712 cases found that new thromboembolic complications, peripheral septic lesions, and even death were more prevalent in patients with delayed diagnosis.^9^

The treatment standard is currently intravenous antibiotics with anaerobic coverage, usually β-lactam and metronidazole.^3^ The recommended duration of treatment is 3–6 weeks, including the transition to oral therapy.^3^ The role of anticoagulation remains controversial, and its use is deferred to physician evaluation in each case, with particular attention to those with extensive thrombosis or persistent septic emboli.^5^ Surgical drainage for abscess is occasionally done and is reserved for atypical cases.^10^ Considering the risks and nonspecific presentation, this case illustrates POCUS as a frontline diagnostic tool in Lemierre syndrome.

## CASE REPORT

A 58-year-old woman with a history of chronic obstructive pulmonary disease, migraines, and recent dental extractions presented to the emergency department with two days of progressively worsening right-sided neck pain and swelling. She denied systemic symptoms such as fever, chills, or recent upper respiratory infections. However, she did note an unexplained lung opacity seen on a recent shoulder radiograph, which was currently undergoing monitoring by her primary care physician. Physical examination revealed a tender, swollen anterior neck mass with mild pharyngeal erythema but no airway compromise or neurologic deficits.

**Image 1:**
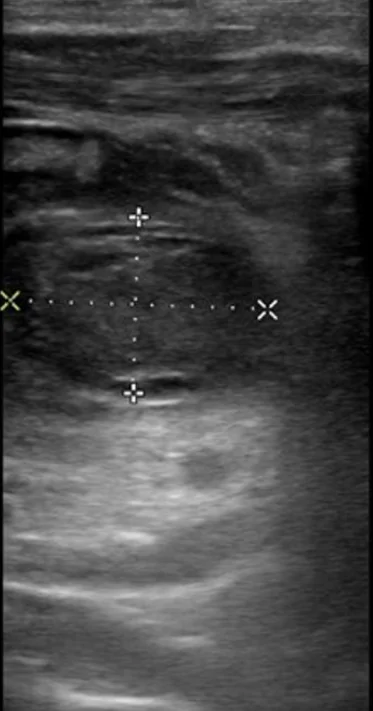
Ultrasound measurement of the internal jugular vein demonstrating a 1.22 × 1.80 centimeters intraluminal hyperechoic thrombus in a 58-year-old woman who presented to the emergency department with two days of progressively worsening right-sided neck pain and swelling.

**Image 2:**
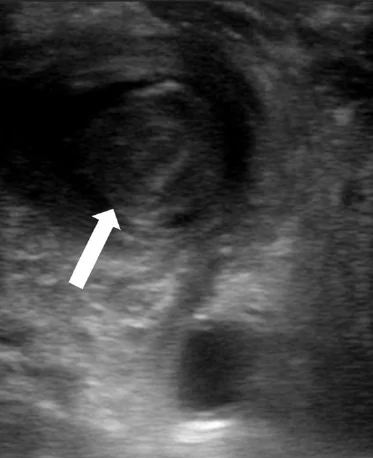
Close-up ultrasound image highlighting the internal jugular vein thrombus (arrow) in a patient diagnosed with Lemierre syndrome.

A POCUS examination of the neck revealed a 1.22 × 1.80 cm hyperechoic mass in the right internal jugular vein with surrounding cobblestone edema (Images 1 and 2).

These findings were consistent with Lemierre syndrome, a type of septic thrombophlebitis specific to the internal jugular vein (Video). The external jugular vein and carotid artery appeared normal. The patient was admitted to the hospital and further diagnostic testing confirmed her initial diagnosis.

Laboratory results, including complete blood count, complete metabolic panel, and liver function tests, were within normal limits. Blood cultures were drawn to assess anaerobic pathogens, although the patient was given empiric intravenous beta lactamase-resistant antibiotics prior to blood culture results, with coverage tailored toward anaerobes. The blood cultures returned later as negative, displaying no growth. She was discharged on hospital day four and has had no further complications to date.

*CPC-EM Capsule*

What do we already know about this clinical entity?

*Lemeirre syndrome is a rare but severe thrombophlebitis of the internal jugular vein, most often presenting after oropharyngeal infection.*

What makes this presentation of disease reportable?

*This case report highlights point-of-care ultrasound (POCUS) findings in still image and video format, demonstrating results in real time.*

What is the major learning point?

*POCUS is a versatile imaging modality that can expedite diagnosis in patients with suspected Lemierre syndrome.*

How might this improve emergency medicine practice?

*Expedited diagnosis of Lemierre syndrome using POCUS could significantly decrease the chance of poor outcomes such as septic shock and Intensive Care Unit admission.*

## DISCUSSION

Lemierre syndrome presents a diagnostic challenge in the ED due to its rarity and nonspecific symptoms in the early stages.^2^,^3^,^11^ Although CT remains the gold standard for diagnosis, POCUS offers real-time vascular assessment and may provide earlier recognition, as demonstrated in this case.^4^,^12^,^13^ Point-of-care ultrasound shows high specificity and sensitivity to detect internal jugular vein thrombosis when performed by trained emergency physicians.^5^,^6^,^14^ It offers a low-cost, noninvasive, and rapid alternative when CT is not immediately available or when radiation exposure is a concern.^5^,^6^,^12^ Additionally, POCUS can help monitor the resultant treatment by evaluating the progression of the internal jugular vein thrombus.^11^ In this case, early bedside identification allowed for prompt initiation of antibiotics, preventing further complications such as sepsis, persistent emboli, and metastatic abscesses.^11^,^15^

## CONCLUSION

Lemierre syndrome is a rare, life-threatening condition for which early diagnosis is central to improving patient outcomes. Early symptoms are nonspecific and can be easily overlooked. This case underscores the importance of point-of-care ultrasound in rapid diagnosis in the emergency department.

## Supplementary Information

Video Point-of-care ultrasound neck examination. The anatomical structures of the internal jugular vein (white dotted circle) and carotid artery (red asterisk) are prominent. The thrombus within the internal jugular vein (red shadow) and cobblestone edema (yellow arrows), indicative of Lemierre syndrome, can also be seen.

## References

[1] Allen BW, Anjum F, Bentley TP Lemierre syndrome. StatPearls [Internet].

[2] Eilbert W, Singla N (2013). Lemierre’s syndrome. Int J Emerg Med.

[3] Carius BM, Koyfman A, Long B (2022). High risk and low prevalence diseases: Lemierre’s syndrome. Am J Emerg Med.

[4] Gaillard F, Bell D, Di Muzio B Lemierre syndrome.

[5] Lee WS, Jean SS, Chen FL (2020). Lemierre’s syndrome: a forgotten and re-emerging infection. J Microbiol Immunol Infect.

[6] Agonafir DB, Diress AE, Saleh AA (2025). Lemierre syndrome: a case report and literature review on atypical presentation. Medicine (Baltimore).

[7] Castro-Marín F, Kendall JL (2010). Diagnosis of Lemierre syndrome by bedside emergency department ultrasound. J Emerg Med.

[8] Nygren D, Holm K (2020). Invasive infections with *Fusobacterium necrophorum* including Lemierre’s syndrome: an 8-year Swedish nationwide retrospective study. Clin Microbiol Infect.

[9] Valerio L, Zane F, Sacco C (2021). Patients with Lemierre syndrome have a high risk of new thromboembolic complications, clinical sequelae and death: an analysis of 712 cases. J Intern Med.

[10] Righini CA, Karkas A, Tourniaire R (2014). Lemierre syndrome: study of 11 cases and literature review. Head Neck.

[11] Gudinchet F, Maeder P, Neveceral P, Schnyder P (1997). Lemierre’s syndrome in children: high-resolution CT and color Doppler sonography patterns. Chest.

[12] Craven P, End B, Griffin P (2023). Emergency department point-of-care ultrasound identification of suspected Lemierre’s syndrome: a case report. Clin Pract Cases Emerg Med.

[13] Davies O, Than M (2012). Lemierre’s syndrome: diagnosis in the emergency department. Emerg Med Australas.

[14] Weeks DF, Katz DS, Saxon P, Kubal WS (2010). Lemierre syndrome: report of five new cases and literature review. Emerg Radiol.

[15] Shook J, Trigger C (2014). Lemierre’s syndrome. West J Emerg Med.

